# Clinicopathological correlations of mesenteric fibrosis and evaluation of a novel biomarker for fibrosis detection in small bowel neuroendocrine neoplasms

**DOI:** 10.1007/s12020-019-02107-4

**Published:** 2019-10-09

**Authors:** Faidon-Marios Laskaratos, Dalvinder Mandair, Andrew Hall, Sarah Alexander, Conrad von Stempel, Josephine Bretherton, TuVinh Luong, Jennifer Watkins, Olagunju Ogunbiyi, Krista Rombouts, Martyn Caplin, Christos Toumpanakis

**Affiliations:** 1 Neuroendocrine Tumour Unit, Centre for Gastroenterology, ENETS Centre of Excellence, Royal Free London NHS Foundation Trust and University College London, London, UK; 2 Department of Cellular Pathology, Royal Free London NHS Foundation Trust, London, UK; 3 Department of Radiology, Royal Free London NHS Foundation Trust, London, UK; 4 Department of Colorectal Surgery, Royal Free London NHS Foundation Trust, London, UK; 5 Regenerative Medicine & Fibrosis Group, Institute for Liver and Digestive Health, Division of Medicine, University College London, London, UK

**Keywords:** Neuroendocrine tumour, Biomarker, NETest, Fibrosis

## Abstract

**Purpose:**

Mesenteric fibrosis (MF) in small intestinal neuroendocrine neoplasms (SINENs) is often associated with significant morbidity and mortality. The detection of MF is usually based on radiological criteria, but no previous studies have attempted a prospective, multidimensional assessment of mesenteric desmoplasia to determine the accuracy of radiological measurements. There is also a lack of non-invasive biomarkers for the detection of image-negative MF.

**Methods:**

A multidimensional assessment of MF incorporating radiological, surgical and histological parameters was performed in a prospective cohort of 34 patients with SINENs who underwent primary resection. Pre-operative blood samples were collected in 20 cases to evaluate a set of five profibrotic circulating transcripts—the “fibrosome”—that is included as an “omic” component of the NETest.

**Results:**

There was a significant correlation between radiological and surgical assessments of MF (*p* < 0.05). However, there were several cases of image-negative MF. The NETest-fibrosome demonstrated an accuracy of 100% for the detection of microscopic MF.

**Conclusions:**

The detection of MF by radiological criteria has limitations. The NETest-fibrosome is a promising biomarker for fibrosis detection and further validation of these results would be needed in larger, multicentre studies.

## Introduction

The development of mesenteric fibrosis (MF) in small intestinal neuroendocrine tumours (SI NETs) is associated with significant morbidity [1, 2] and may also adversely affect patient prognosis [3–5]. Despite its sinister and substantial clinical ramifications, MF remains an under-researched area of neuroendocrine neoplasia and its pathophysiology is poorly understood [1].

Typically, the presence of mesenteric desmoplasia is determined radiologically. However, the assessment of MF is a problematic area, because there is very limited literature on the multidimensional evaluation of fibrosis using a triangulation of different methodologies. To the best of our knowledge, the present study is the first report of a prospective evaluation of mesenteric desmoplasia using different methods of assessment. Our hypothesis was that conventional imaging may have limitations particularly for the detection of small amounts of fibrosis that can be revealed by histological examination of the mesenteric mass. Although histological measures of MF are not routinely used, we decided to evaluate two parameters: (1) the width of fibrous bands, which was used in an older study of MF [6] and (2) the Collagen Proportionate Area (CPA), which has been previously used in the field of hepatology as an index of liver fibrosis severity [7].

Based on our hypothesis that clinical assessments of MF may not necessarily detect minimal degrees of fibrosis evident at histological level, we decided to also evaluate a non-invasive biomarker with potential utility for the detection of ‘image-negative’ desmoplasia. Currently, there is a lack of clinically useful biomarkers for fibrosis in SI NETs. Although several non-invasive biomarkers have been investigated in the context of carcinoid heart disease [1], only a few studies have assessed the utility of non-invasive biomarkers (serum CTGF [Connective Tissue Growth Factor], urinary 5-HIAA [5-hydroxyindoleacetic acid]) in MF [3, 8, 9]. However, these biomarkers have modest performance metrics, require further validation and have not gained acceptance.

The NETest is a PCR-based tool that measures a panel of 51 circulating transcripts and has an excellent sensitivity and specificity for the diagnosis of neuroendocrine neoplasia [10, 11]. We hypothesised that a subset of genes within this 51-gene panel with defined roles in fibrosis development—the fibrosome—may be predictive of mesenteric desmoplasia.

Therefore, the main aims of this prospective study were firstly to evaluate MF using a multidimensional approach and assess the accuracy of radiological criteria, and secondly to evaluate the performance of the NETest-fibrosome panel as a blood-based biomarker for the detection of MF.

## Materials and methods

A total of 34 patients with SI NETs, who underwent primary resection at the Royal Free Hospital, ENETS Centre of Excellence between 2016 and 2018, were prospectively recruited into this study. Informed consent was obtained from each patient included in the study. The study protocol conforms to the ethical guidelines of the 1975 Declaration of Helsinki (6th revision, 2008) as reflected in a priori approval by the institution’s human research committee (UCL Biobank Ethical Review Committee approval [reference number NC2017.003]). A summary of patient characteristics is provided in Table 1.

**Table 1:** Summary of demographic and clinical characteristics of patients enrolled in the desmoplasia evaluation study

	Patients with midgut NETs who underwent surgery (*n* = 34) *n* (%)
Age (mean ± SD, years)	61 ± 13
Sex	
Male	23 (68%)
Female	11 (32%)
Grade	
1	21 (62%)
2	13 (38%)
Extent of disease	
Localised	3 (9%)
Locoregional	9 (26%)
Metastatic	22 (65%)
Mesenteric mass	31 (91%)
Liver metastases	17 (50%)
Distant extrahepatic metastases	10 (29%)
Macroscopic mesenteric fibrosis	25 (74%)
Medical therapy	
Octreotide LAR	10 (29%)
Lanreotide Autogel	8 (24%)
Surgical therapy	
Small bowel resection	1 (3%)
Right hemicolectomy (R0)	24 (71%)
Right hemicolectomy (R1)	9 (26%)

A multidimensional assessment of MF was used, and the following components were assessed:

(i) The *radiological* severity of mesenteric desmoplasia was based on the scoring system originally proposed by Pantongrag-Brown et al. [6] using the following categories: (a) No radiological evidence of mesenteric desmoplasia (Absence of radiating strands), (b) Mild desmoplasia (≤10 thin radiating strands), (c) Moderate desmoplasia (>10 thin strands or <10 thick strands) and (d) Severe desmoplasia (≥10 thick strands).

(ii) The *histological* assessment of MF was based on the histological slide with the maximum amount of fibrous tissue. Surgical resection specimens (rather than biopsy material) were used for this purpose to minimise the risk of sampling error. In fibrotic patients the mesenteric mass and surrounding tissue were examined for fibrous tissue using Sirius Red staining, while in non-fibrotic patients (who did not have a mesenteric mass), the non-fibrotic mesentery adjacent to the resected primary tumour was examined. The histological slide was stained with a connective tissue stain (Sirius Red) and two parameters were measured:

(a) The width of the thickest fibrous band surrounding the tumour. This technique was used previously by Pantongrag-Brown et al. and showed a correlation with the radiological assessment of MF [6]. In their publication, Pantongrag-Brown et al. also introduced a new histological parameter, the so-called *‘fibrosis grade’*, which is based on the maximum width (*grade 1*: width < 1 mm, *grade 2*:1–2 mm, *grade 3*: >2 mm) [6].

(b) The CPA, which represents the percentage of collagen in the stroma surrounding the tumour. This is a quantitative method of measuring fibrous tissue using digital image analysis and has been validated in liver cirrhosis [7, 12].

### Optimisation/characterisation of the inter-observer variability

The cross-sectional imaging (CT/MRI scan) was assessed independently by two assessors (CS and JB) with good inter-observer agreement. In a small number of cases (*n* = 3) a minor discrepancy was observed between the two assessments and consensus was reached between the assessors after a final review of the imaging studies.

The histological slides were assessed independently by two assessors (AH and SA) with good inter-observer variability. In the case of minor discrepancies (<20% difference between the two measurements) the mean value of the two assessments was calculated and used for our analysis. In the small number of cases with more significant discrepancies (>20% difference between the two measurements), consensus was reached between the two assessors after a final review of the slides.

(iii) A ***surgical*** assessment of the extent of MF in relation to the entire small bowel mesentery was also provided using the following categories: (a) No desmoplasia (No MF), (b) Mild desmoplasia (MF involving <25% of the small bowel mesentery), (c) Moderate desmoplasia (MF involving 25–50% of the small bowel mesentery) and (d) Severe desmoplasia (MF involving >50% of the small bowel mesentery).

This assessment was provided by the operating surgeon (the same surgeon [OO] performed the macroscopic assessment of mesenteric desmoplasia in all the cases).

A total of 20 patients were included in the biomarker assessment study (a subset of 19 patients from the mesenteric desmoplasia evaluation study and an additional patient who had extensive MF and unresectable disease, who was not included in the desmoplasia assessment study, since no histology was available). The characteristics of this patient cohort are summarised in **Online Resource 1**. No patients had carcinoid heart disease or other fibrotic conditions.

The presence of MF was assessed using a multidimensional approach, incorporating radiological, surgical and histological parameters. We then evaluated the utility of the Fibrosome in the detection of both macroscopic and microscopic fibrosis. Two patients were classified as ‘non-fibrotic’ and eighteen as ‘fibrotic’.

A total of 31 blood samples were collected pre-operatively (within 24 h of surgery) in 5 ml EDTA tubes and stored in −80 °C within 2 h of collection (samples immediately stored on ice/4 °C after sampling). De-identified samples were shipped on dry ice to Wren laboratories, USA for analysis. The methodology of NETest measurements has been previously described [13–15].

For this study, we assessed a subset of five circulating transcripts (from the entire 51-gene molecular signature) with known roles in fibrosis, namely: *CTGF*, *CD59*, *APLP2* (amyloid precursor-like protein 2), *FZD7* (frizzled homologue 7) and *BNIP3L*. These five genes (with the exception of CTGF) have not been investigated in the context carcinoid-driven fibrosis but have been linked to fibrosis in other conditions. **FDZ (Frizzled)** are seven-transmembrane receptors that bind Wnt proteins and mediate the canonical and non-canonical Wnt signalling pathways. Wnt signalling plays important roles in tissue development and repair, as well as carcinogenesis, but more recently it has also been implicated in fibrogenesis [16, 17]. FDZ7 in particular has been shown to mediate TGFβ-induced pulmonary fibrosis via the non-canonical Wnt signalling pathway and lead to the expression of collagen I, fibronectin, CTGF and α-SMA in lung fibroblasts [18]. **CTGF** is a known mediator of fibrosis, which acts downstream of TGFβ, and has been previously investigated in carcinoid-related desmoplasia and other fibrotic conditions [1, 9, 19]. **BNIP3L** is also implicated in cardiac fibrosis, where it is known to promote TGFβ expression in cardiac fibroblasts [20]. Moreover, **CD59** is a regulator of complement activation and inhibits the formation of the membrane attack complex. The complement system is involved not only in innate immunity and adaptive responses but also in tissue repair and fibrosis [21, 22]. Thus, CD59 may be viewed as a regulator of fibrosis. Finally, **APLP2** is widely expressed in human cells and has been implicated in cancer progression. A recent study in Drosophila demonstrated that APLP2 expression promotes cell migration by inducing matrix metalloproteinase MMP1 expression, which in turn leads to basement membrane degradation [23]. Therefore, this protein may play a role in extracellular matrix remodelling and its precise role in carcinoid-related fibrosis needs to be further investigated.

Statistical analysis was performed using GraphPad Prism® version 8 and SPSS version 25 statistical software. A *p*-value < 0.05 was considered statistically significant.

## Results

### Inter-observer variability

There was a 91% agreement in the radiological assessment of mesenteric desmoplasia between the two assessors. In addition, the inter-observer variability in the histological measurements was very small for both the CPA (Spearman’s correlation *r* = 0.86998 [95% CI 0.7487, 0.9347], *p* < 0.0001) and width of fibrous band measurements (Spearman’s correlation *r* = 0.9174 [95% CI 0.8366, 0.9591], *p* < 0.0001) between the two assessors.

### Correlation of surgical and radiological assessments of MF

There was a statistically significant correlation between the surgical and radiological methods of assessment of MF (Fisher’s exact test, *p* = 0.014) (Table 2). Of those patients without the evidence of MF on cross-sectional imaging (*n* = 15), no fibrous tissue was detected intra-operatively in nine cases (60%), while the remaining patients (40%) had fibrosis macroscopically. On the other hand, when fibrosis was detected radiologically (*n* = 19), this was also seen intra-operatively in most cases (*n* = 16) (84%) (Table 2).

**Table 2:** Correlation of radiological and surgical assessments of mesenteric desmoplasia (Fisher’s exact test, *p* = 0.014)

		Surgical				Total
		None	Mild	Moderate	Severe	
Radiological	None	9	5	1	0	15
	Mild	3	2	1	0	6
	Moderate	0	7	1	1	9
	Severe	0	4	0	0	4
Total		12	18	3	1	34

### Correlation of surgical/radiological and histological assessments of MF

**Fig. 1:**
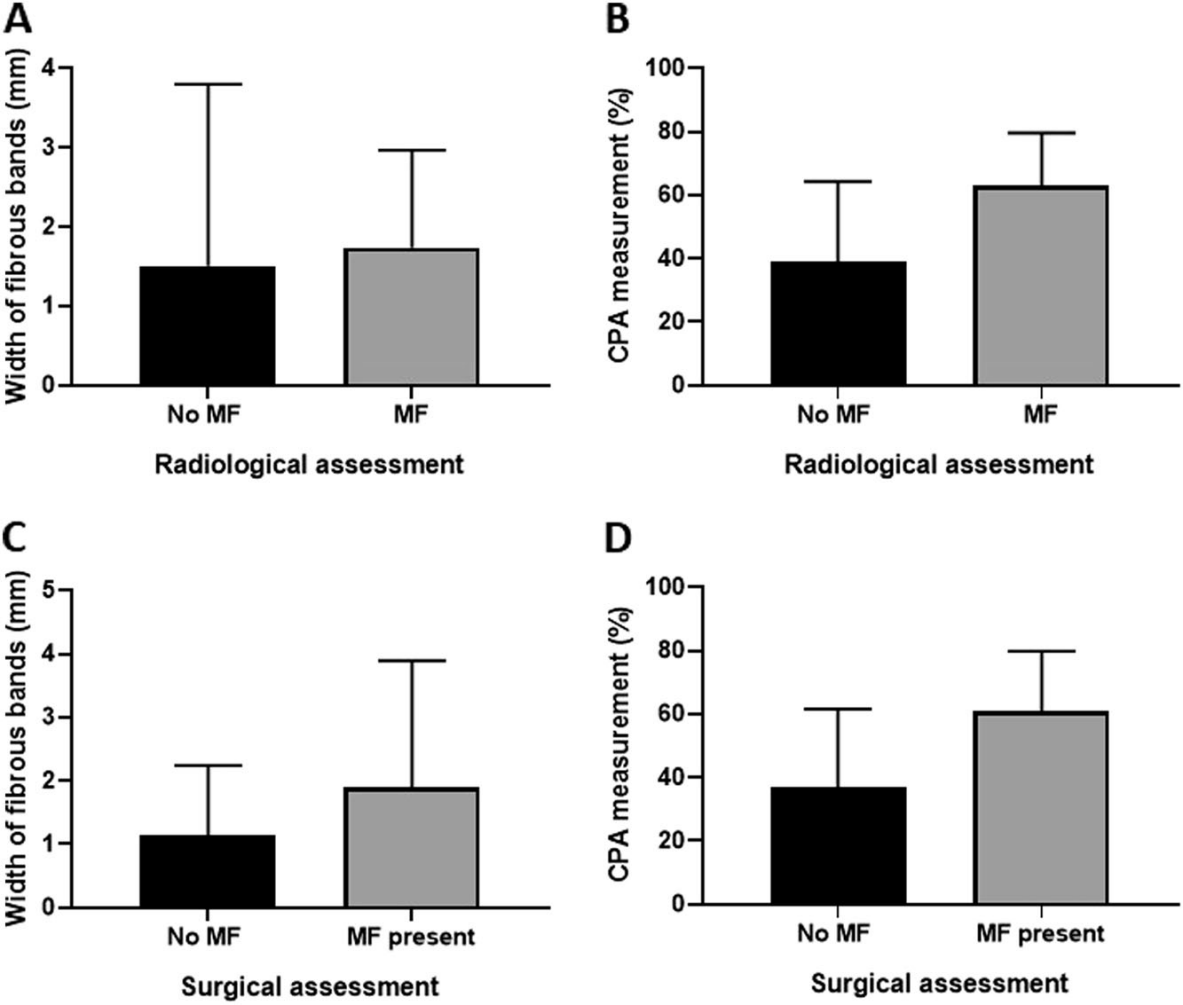
Correlation of the presence of MF by radiological/surgical criteria with histological measurements of fibrosis. In several cases, there was histological evidence of fibrosis which was not seen on imaging studies or intra-operatively

**Fig. 2:**
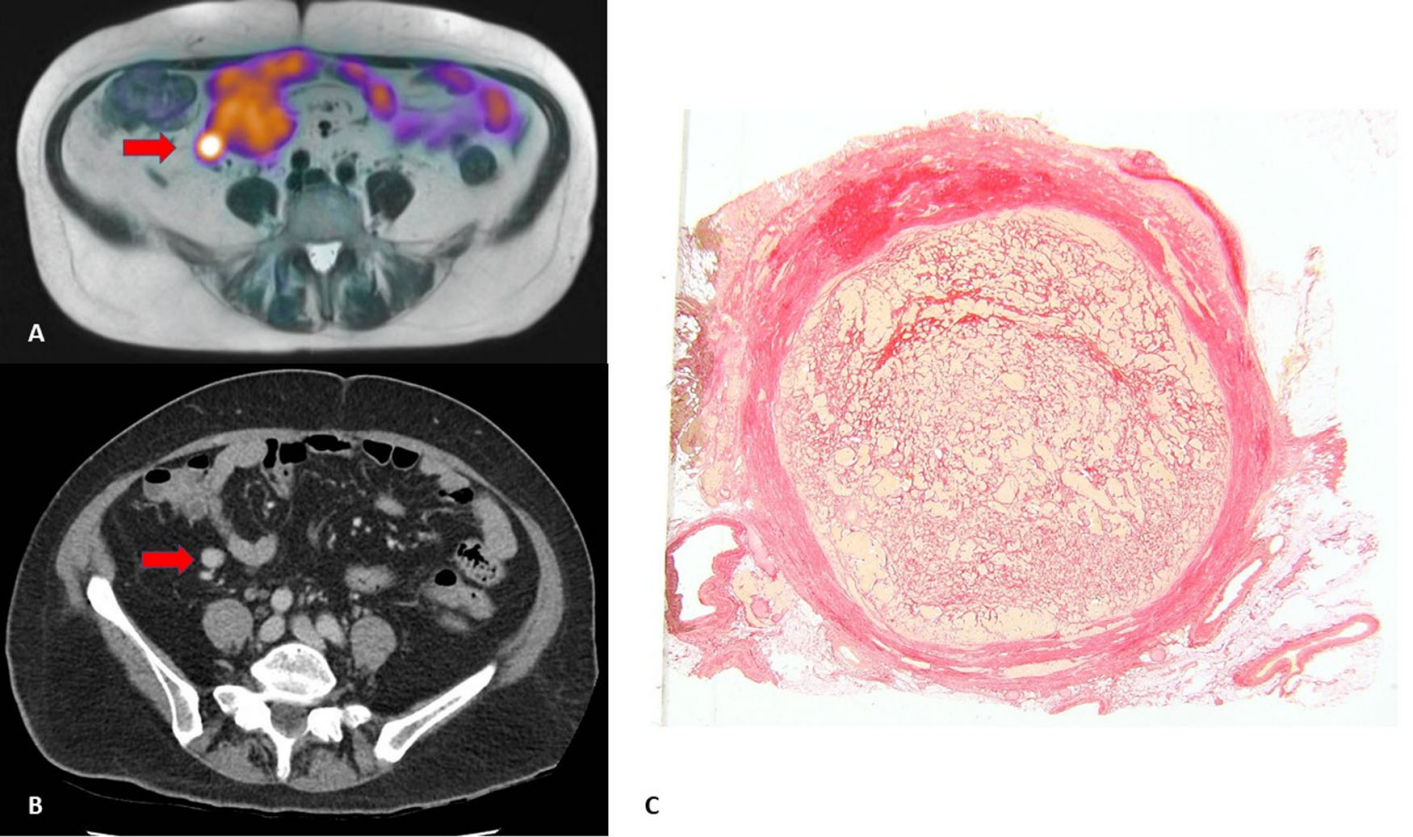
Correlation of ^68^Ga PET/MRI (**a**), CT abdomen (**b**) and histology (**c**) in a patient with a SI NET. (a + b) A gallium-avid mesenteric mass is seen without surrounding desmoplasia on imaging. **c** However, on histology a fibrotic capsule is seen surrounding the mesenteric lymph node, indicating the presence of image-negative mesenteric desmoplasia

**Fig. 3:**
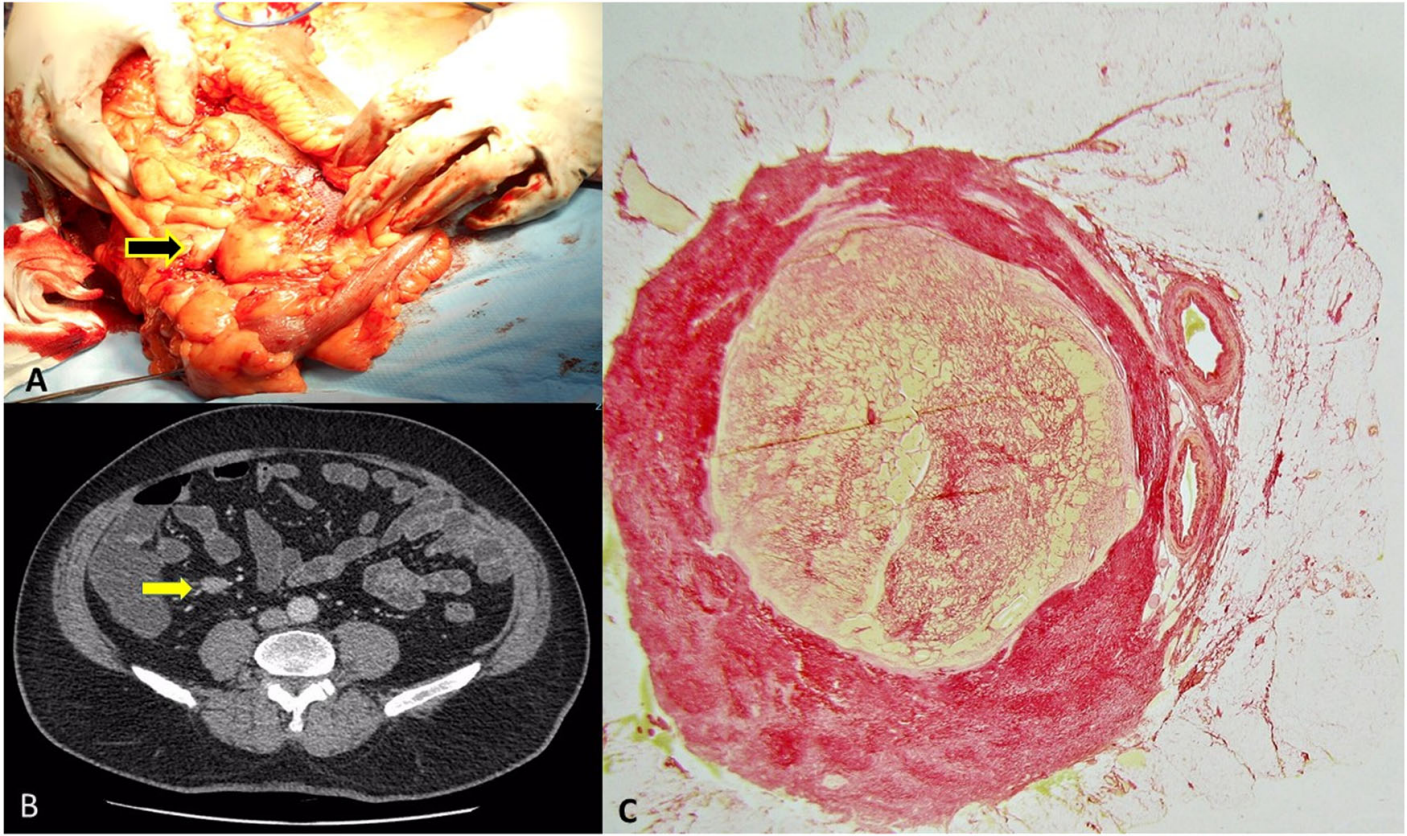
Review of surgical (**a**), radiological (**b**) and histological (**c**) assessments in a patient with a SI NET. **a** The primary tumour and mesenteric lymph node were removed laparoscopically. A small, soft palpable lymph node was seen intra-operatively with no obvious surrounding fibrosis. **b** Similarly, the CT scan showed a small lymph node with some subtle spiculation, but no evident desmoplasia with the typical ‘stellate pattern’. **c** The histological slide of the lymph node with Sirius red staining showed a fibrotic capsule around the small (~14 mm) metastatic lymph node. This minimal amount of fibrous tissue was not obvious at macroscopic assessments

In several cases there was histological evidence of fibrosis around the mesenteric mass, which was not seen radiologically, indicating the presence of image-negative mesenteric desmoplasia (Figs. 1–3). Similarly, often MF was present histologically but not detected intra-operatively by macroscopic inspection (Figs 1 and 3)

### Evaluation of the NETest-fibrosome as a biomarker for MF

In this small cohort of 20 patients there was one patient who did not appear to have obvious mesenteric desmoplasia at macroscopic assessments of fibrosis, although some minimal fibrosis was detected histologically (Fig. 3). In this case, a thin fibrous capsule was seen around a small mesenteric lymph node. Although the natural history of mesenteric mass formation is not well documented in the literature, this small fibrotic lymph node would conceivably have developed into a larger fibrotic mesenteric mass, if it had been left in situ. Therefore, the ability of a biomarker to detect both macroscopic and microscopic fibrosis may be of clinical utility in anticipating the development of fibrosis, when this is not evident using solely macroscopic assessments.

Patients with macroscopic and microscopic MF were included in the fibrotic group and the ability of the five circulating transcripts from the NETest (APLP2, BNIP3L, CD59, CTGF and FZD7) to define a fibrotic phenotype was assessed.

**Fig. 4:**
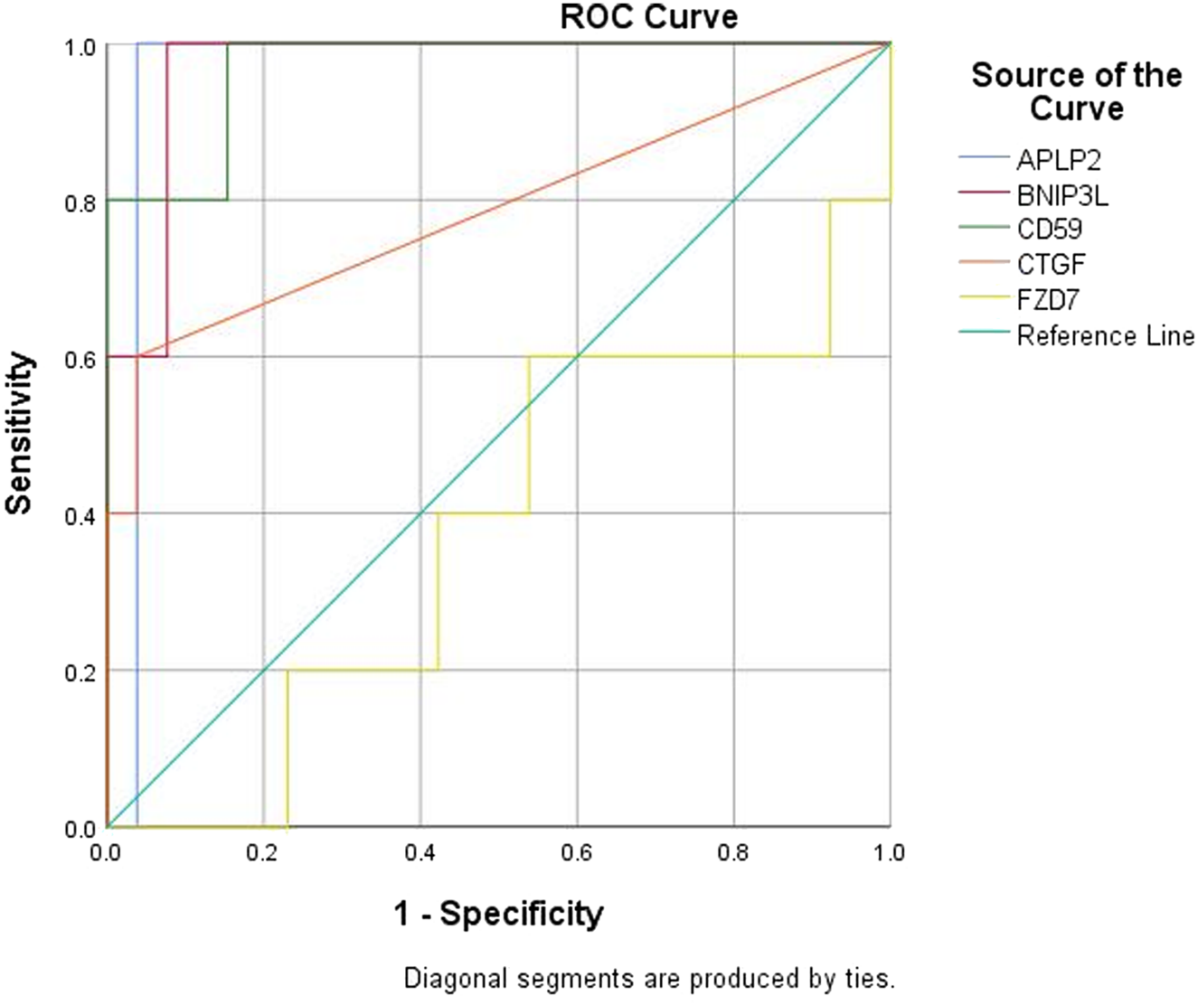
Receiver Operating Characteristic (ROC) Curve analysis demonstrating the ability of five NETest-fibrosome genes (APLP2, BNIP3L, CD59, CTGF and FZD7) to define a fibrotic phenotype by microscopic criteria

ROC curve analysis demonstrated that four circulating transcripts (APLP2, BNIP3L, CD59 and CTGF) could independently predict the presence of MF at a statistically significant level. More specifically, the metrics for each individual transcript were the following: APLP2 (AUC 0.962, 95% CI 0.888, 1.000, *p* = 0.001), BNIP3L (AUC 0.969, 95% CI 0.912, 1.000, *p* = 0.001), CD59 (AUC 0.969, 95% CI 0.904, 1.000, *p* = 0.001), CTGF (AUC 0.785, 95% CI 0.510, 1.000, *p* = 0.047) and FZD7 (AUC 0.377, 95% CI 0.095, 0.659, *p* = 0.390) (Fig. 4).

The mathematical combination of the five circulating transcripts achieved an AUC of 1.000 (95% CI 1.000, 1.000, *p* < 0.001) and a predictive model based on the combination of these transcripts exhibited an accuracy of 100% for predicting the presence of MF (sensitivity 100%, specificity 100%) (Table 3). This demonstrated the ability of these five circulating transcripts to determine the presence of desmoplasia, not only when it was macroscopically evident but also when it was detected only histologically.

**Table 3:** Accuracy of a predictive model utilising five circulating transcripts from the NETest (APLP2, BNIP3L, CD59, CTGF and FZD7) in predicting the presence of microscopic mesenteric fibrosis

Observed	Predicted		
	Fibrosis		Percentage correct
	F	NF	
Fibrosis			
F	26	0	100.0
NF	0	5	100.0
Overall Percentage			100.0

*F* fibrotic group, *NF* non-fibrotic group

## Discussion

The present study is the first report of a prospective correlation of surgical, radiological and histopathological findings of MF associated with SI NETs and also the first study evaluating a circulating transcriptomic signature as a biomarker for the prediction of MF.

It is quite surprising that we could identify only one old, small, retrospective study published by Pantongrag-Brown et al. nearly 25 years ago, that evaluated the severity of MF by both radiological and histological criteria in midgut NETs with a mesenteric mass. Interestingly, in this study 21 cases with an associated mesenteric mass were evaluated by both methods (computed tomography and histology) and fibrotic tissue was detected histologically in all those cases [6]. This is in keeping with our observations that suggest that the presence of a mesenteric mass was invariably associated with the development of fibrosis, although sometimes this was not detected on imaging and was only seen histologically as a ‘fibrous capsule’ (Figs 1 and 3). This is a quite unusual pattern which has not been reported previously and is distinct to the typical ‘stellate’ or ‘spoke-wheel’ appearance, which is described in the literature as a pathognomonic sign of a midgut carcinoid with associated mesenteric desmoplasia. Thus, our data demonstrate clearly that cross-sectional imaging often underestimates the presence of fibrosis. Although the clinical significance of image-negative MF is currently unknown (since this is a new concept and no previous studies have assessed the evolution of this condition), conceivably minimal fibrosis can progress over time and in some cases become advanced and lead to complications, if it remains unrecognised and is left in situ. Thus, our better understanding and recognition of image-negative desmoplasia may be important not only for clinical purposes but also because it will hopefully allow the investigation of the natural history of this entity in future studies.

Although our study showed that macroscopic assessments (radiological or surgical) of MF were often inaccurate and therefore histological measurements should be the gold standard for the determination of MF, the most significant limitation of histological measures is that a surgical resection specimen is required. Therefore, the development of circulating biomarkers with a high sensitivity and specificity for the pre-operative detection of image-negative mesenteric desmoplasia may have important clinical utility.

In the present study we evaluated a subset of five genes from the NETest that are related to fibrosis and assessed their performance metrics in the detection of macroscopic and microscopic fibrosis. The NETest is a PCR-based 51 transcript signature that has an excellent (>90%) sensitivity and specificity for the diagnosis of gastroenteropancreatic NETs, and has been shown to outperform conventional secretory biomarkers, such as chromogranin A [11, 13, 14, 24, 25]. In addition, this molecular signature correlates with disease status [26, 27] and captures the hallmarks of neuroendocrine neoplasia [28]. The NETest has also been shown to predict response to somatostatin analogue therapy [15], peptide receptor radionuclide therapy [29, 30], operative resection and ablation strategies [31].

Given the ability of this multianalyte to act as a liquid biopsy that can capture the multidimensionality of neuroendocrine neoplasia, we hypothesised that a subset of five genes from the NETest (APLP2, BNIP3L, CTGF, CD59 and FDZ7) that are involved in fibrosis—the fibrosome—may be a clinically useful and accurate biomarker of MF. In this small cohort of 20 patients, who did not have carcinoid heart disease or other fibrotic disorders, the fibrosome could accurately predict the presence of microscopic (image-negative) fibrosis (100%). This mirrors the ability of circulating transcripts (NETest) to detect microscopic tumour burden, when conventional imaging modalities (CT/MRI and ^68^Ga PET/CT) are negative (image-negative liver disease) [32], although the clinical implication of such micro-metastatic disease and its impact on medical management strategies remain unclear.

There are several limitations to the present study. Firstly, the number of patients is relatively small and therefore validation of our findings in larger, ideally multicentre, prospective studies would be needed. Secondly, the radiological evaluation of MF included CT imaging in some cases and MRI in others, and although no studies have compared the sensitivity and specificity of these different techniques in fibrosis detection, this may have led to some discrepancies in these evaluations. Also, recent advances in imaging modalities mean that a direct comparison with the older study of Pantongrag-Brown et al. published in 1995 may not be entirely valid. However, this is the only study in the literature where such clinico-pathological evaluations of MF were performed. Thirdly, the surgical assessments of MF were rather subjective and based on a macroscopic evaluation during surgery when an accurate assessment can sometimes be difficult (for example, in the context of bleeding). Finally, we assessed a circulating molecular signature as a biomarker for fibrosis. Conceivably the levels of circulating transcripts in the blood might be affected not only by the levels of gene expression in the tissue, but also the size of the primary tumour and fibrotic mesenteric mass, as well as treatments (e.g. somatostatin analogues), and although this is currently not known, it should be mentioned as a potential limitation.

In conclusion, this study has utilised a triangulation of different methodologies to assess MF in SI NETs and has introduced the concept of image-negative mesenteric desmoplasia. It has also investigated the role of a novel circulating biomarker in the detection of MF. In future studies, these findings would need to be externally validated in additional and larger patient cohorts. Furthermore, the clinical role of this circulating molecular signature in other fibrotic complications of neuroendocrine tumours (such as carcinoid heart disease) would need to be explored, as well as its specificity for carcinoid-related fibrosis in patients with other fibrotic conditions. These studies will define the role of this promising novel biomarker and delineate its clinical utility in a variety of clinical applications.

## Supplementary information

Supplementary Information
