# Supplementary material for: Clinicopathological correlations of mesenteric fibrosis and evaluation of a novel biomarker for fibrosis detection in small bowel neuroendocrine neoplasms
Source: Endocrine. 2019 Oct 9;67(3):718–26. doi: 10.1007/s12020-019-02107-4 (PMC7054371; doi:10.1007/s12020-019-02107-4)
Supplement: Supplementary file 1 — Supplementary Information [file 12020_2019_2107_MOESM1_ESM.pdf]

Article Title: Clinicopathological correlations of mesenteric fibrosis and evaluation of a novel biomarker for fibrosis detection in small bowel neuroendocrine neoplasms

Journal: Endocrine

Authors: Faidon-Marios Laskaratos, Dalvinder Mandair, Andrew Hall, Sarah Alexander, Conrad von Stempel, Josephine Bretherton, TuVinh Luong, Jennifer Watkins, Olagunju Ogunbiyi, Krista Rombouts, Martyn Caplin, Christos Toumpanakis

Corresponding author: Dr Faidon-Marios Laskaratos MD, MSc, MRCP, Royal Free London NHS Foundation Trust, flaskaratos@gmail.com

Online Resource 1. Summary of demographic and clinical characteristics of patients enrolled in the biomarker assessment study

	Patients with midgut NETs who underwent surgery (n=20)<br>n (%)
**Age (mean±SD)**	63±10
**Sex**	
Male	13 (65%)
Female	7 (35%)
**Grade**	
1	13 (65%)
2	7 (35%)
**Extent of disease**	
Localised	2 (10%)
Locoregional	5 (25%)
Metastatic	13 (65%)
**Mesenteric mass**	18 (90%)
**Liver metastases**	10 (50%)
**Distant extrahepatic metastases**	5 (25%)
**Macroscopic mesenteric fibrosis**	17 (85%)
**Medical therapy**	
Octreotide LAR	6 (30%)
Lanreotide Autogel	7 (35%)
**Surgical therapy**	
Small bowel resection	1 (5%)
Gastro-jejunal bypass	1 (5%)

Right hemicolectomy (R0)<br><br>Right hemicolectomy (R1)	10 (50%)<br><br>8 (40%)
